# Phase matters: A role for the subthalamic network during gait

**DOI:** 10.1371/journal.pone.0198691

**Published:** 2018-06-06

**Authors:** Gabriele Arnulfo, Nicolò Gabriele Pozzi, Chiara Palmisano, Alice Leporini, Andrea Canessa, Joachim Brumberg, Gianni Pezzoli, Cordula Matthies, Jens Volkmann, Ioannis Ugo Isaias

**Affiliations:** 1 Department of Neurology, University Hospital and Julius-Maximillian-University, Wuerzburg, Germany; 2 Department of Informatics, Bioengineering, Robotics and System Engineering, University of Genoa, Genoa, Italy; 3 Department of Electronics, Information and Bioengineering, MBMC Lab, Politecnico di Milano, Milan, Italy; 4 Fondazione Europea di Ricerca Biomedica (FERB Onlus), Cernusco s/N (Milan), Italy; 5 Department of Nuclear Medicine, University Hospital and Julius-Maximillian-University, Wuerzburg, Germany; 6 Centro Parkinson ASST G. Pini-CTO, Milan, Italy; 7 Department of Neurosurgery, University Hospital and Julius-Maximillian-University, Wuerzburg, Germany; University of California Los Angeles, UNITED STATES

## Abstract

The role of the subthalamic nucleus in human locomotion is unclear although relevant, given the troublesome management of gait disturbances with subthalamic deep brain stimulation in patients with Parkinson’s disease. We investigated the subthalamic activity and inter-hemispheric connectivity during walking in eight freely-moving subjects with Parkinson’s disease and bilateral deep brain stimulation. In particular, we compared the subthalamic power spectral densities and coherence, amplitude cross-correlation and phase locking value between resting state, upright standing, and steady forward walking. We observed a phase locking value drop in the *β-*frequency band (≈13-35Hz) during walking with respect to resting and standing. This modulation was not accompanied by specific changes in subthalamic power spectral densities, which was not related to gait phases or to striatal dopamine loss measured with [^123^I]N-ω-fluoropropyl-2β-carbomethoxy-3β-(4-iodophenyl)nortropane and single-photon computed tomography. We speculate that the subthalamic inter-hemispheric desynchronization in the *β-*frequency band reflects the information processing of each body side separately, which may support linear walking. This study also suggests that in some cases (i.e. gait) the brain signal, which could allow feedback-controlled stimulation, might derive from network activity.

## Introduction

Human gait is a complex motor behavior and requires a constant and coordinated flow of information across functionally specialized brain areas [1]. The locomotor network can be schematically divided into a spinal and a supraspinal component comprising of different cortical areas (including the supplementary motor area, SMA), the basal ganglia, the subthalamic nucleus (STN), the mesencephalic locomotor region (MLR), and the cerebellum [1–4]. At a spinal level, a central pattern generator (CPG) can produce the basic motor pattern for stepping (rhythmic motor activity) autonomously, in the absence of supraspinal and sensory inputs[5]. The selection and modulation of gaits (e.g. initiation, termination, velocity, direction, and spatial orientation) are instead performed at the higher levels of the nervous system [6,7].

Locomotion is controlled by the tuned interplay of such neural circuits and can be studied by assessing their oscillatory activity [8,9]. In particular, the phase synchronization between oscillatory neural circuits is hypothesized to facilitate the efficacy of information exchange between different brain areas and could be particularly relevant for an efficient flow of information in large-scale networks, such as the locomotor network [9–13].

In this framework, the role of the basal ganglia is poorly defined but their involvement in locomotion is clearly suggested by the presence of gait impairment in neurological diseases with altered basal ganglia functioning, such as Parkinson’s disease (PD) [14]. In particular, the STN is an essential node of the supraspinal locomotor network, being connected to the SMA and projecting to the MLR directly and through the basal ganglia output nuclei (i.e. the globus pallidus pars interna [GPi] and the substantia nigra pars reticulate [SNr]) [15]. The STN is active during gait [4], and the modulation of its activity with deep brain stimulation (STN-DBS) impacts gait performances [16].

In PD, the loss of striatal dopaminergic innervation determines an excessive synchronization of subcortical neuronal oscillations to cortical rhythm, thus hindering subcortical motor control processing [17–21]. These functional derangements are usually observed in the STN. Local field potentials (LFPs) recorded in unmedicated PD patients showed an excessive synchronization of the STN neuronal oscillations in the *β-*frequency band (≈13–35 Hz), which extends to connected nuclei (e.g. the GPi and motor cortices) and to the opposite hemisphere [19,22–25]. Such synchronization correlates with the severity of parkinsonian symptoms, and diminishes with dopaminergic medications, deep brain stimulation (DBS) and during movement preparation and execution [26].

Published works have focused on the use of *β*-band activity as a control signal for closed-loop DBS in patients with PD [27,28]. Pursuant to such study, extensive neurophysiological work will be required to describe not only symptom-specific physiomarkers [18,20,22], but especially physiological markers of particular brain states (e.g. gait and sleep) [29]. To this end, under the hypothesis of a direct involvement of the STN during fine-tuning of complex and synchronized bilateral movements [19,30,31], we investigated the subthalamic local neuronal oscillations and inter-hemispheric coupling during gait. We also established whether STN oscillatory changes were related to specific phases of gait, assessed with a kinematic analysis, and influenced by striatal dopaminergic loss, measured with [^123^I]N-*ω*-fluoropropyl-2*β*-carbomethoxy-3*β*-(4-iodophenyl)nortropane (FP-CIT) and single-photon computed tomography (SPECT).

## Methods

### Patients and surgery

We enrolled eight subjects with PD implanted at Julius-Maximilians-University-Hospital Wuerzburg between December 2013 and May 2014 with the Activa PC+S^®^ neurostimulation system (Medtronic, PLC). This system allows therapeutic DBS as well as on-demand recording of the LFPs from the implanted electrodes [32,33]. The Activa PC+S^®^ system and the related hard- and software for programming and readout were provided under a request for application agreement with Medtronic, PLC. The company was not involved in study design, patient selection, data analysis, or reporting of the results.

All patients were diagnosed with PD according to the UK Parkinson Disease Brain Bank criteria [34] and met the inclusion criteria for bilateral STN-DBS [35]. None of them had cognitive decline or mood disturbances as evaluated with standardized rating scales (i.e. Parkinson neuropsychometric dementia assessment, Mattis dementia rating scale, Hamilton depression rating scale and the non-motor symptoms scale). For clinical assessment, patients were evaluated within one month before surgery (pre-DBS) with the United Parkinson’s Disease Rating Scales motor score (UPDRS-III) after overnight (>12 h) withdrawal of all dopaminergic medications (meds-off) and upon receiving 1 to 1.5-times (range 200/50-300/75 mg of levodopa/benserazide) the levodopa-equivalent of the morning dose (meds-on) [36]. After surgery (post-DBS), patients were assessed with the UPDRS-III in four conditions: (i) stimulation off for at least two hours (stim-off); (ii) with bilateral STN stimulation (stim-on) [monopolar stimulation (6/7 subjects); mean amplitude: 3.1 mA (SD: 0.76 mA); pulse width 60 μs; mean frequency 138 Hz (SD 31 Hz)]; (iii) meds-on (as before surgery); (iv) meds-on and stim-on. The therapeutic response to DBS or levodopa was expressed as percentage of improvement according to the formula, adapted from Isaias and coll. [37]:

(a−b)/ax100 (1)

where (a) is meds-off UPDRS-III score and (b) is meds-on UPDRS-III score at pre-DBS or meds-off, stim-on UPDRS-III score at the time of the study. In this study, all subjects were tested at least six months after surgery. At the time of the experiment, all patients were on stable dopaminergic treatment (for at least two months) and chronically stimulated (at least one month with unchanged DBS parameters). The Institutional Review Board of the University Hospital Wuerzburg approved the study and all patients gave written informed consent. The applied methods were conducted in accordance with the Declaration of Helsinki.

The surgical procedure has been previously described [38]. In brief, implantation was performed under local anesthesia using Leksell’s Frame (Elekta, Leksell Stereotaxy System, Stockholm, Sweden). The DBS electrode used was model 3389 (Medtronic, PLC) with four platinum–iridium cylindrical contacts of 1.5 mm each and a contact-to-contact separation of 0.5 mm. Contacts 0 and 8 were the lowermost and contacts 3 and 11 the uppermost (E0-3 refers to right- and E8-11 to the left-hemisphere). The intended coordinates for STN were 12 mm lateral, 2 mm posterior, 4 mm ventral to the mid-commissural point and were adjusted according to individual STN delineation on T2-weighted and SWI images (MAGNETOM Trio, SIEMENS Healthcare, Erlangen, Germany) and with intraoperative microelectrode recordings. Micro- and macro-electrode stimulation and intraoperative CT scan also served to confirm targeting. Postoperative scanning (1 mm slice-thickness CT scan fusion with the pre-operative MRI) confirmed the electrode location. The precise localization within the STN of the active contacts used for chronic stimulation was further confirmed by means of SureTune^TM^ (Medtronic, PLC) [39].

### Protocol, set-up and biomechanical data processing

Recordings were performed at the gait laboratory of the Department of Neurology, University Hospital Wuerzburg. Patients were investigated in the morning at least 12 h after their last dose of antiparkinsonian medication and at two hours after pausing the stimulation. Patients performed at least three trials (range 3–7) according to their clinical conditions for an assessment program involving three conditions: (i) resting (i.e. quietly sitting for 60 s with eyes open during random thinking); (ii) upright standing (for 60 s); (iii) over-ground bare-foot walking at self-selected speed.

For the biomechanical analysis, we considered the steps performed at steady state velocity (steady state linear walking) in a calibrated volume of 8 x 2 x 5 m (total number of 227 strides, range: 19–37 strides per patient). Kinematics of body segments was measured using an optoelectronic system (SMART-DX400, BTS, Milano, Italy), which computed the 3D coordinates of 29 spherical retro-reflective markers (15 mm diameter) fixed to anatomical landmarks [40,41]. The marker coordinates were low-pass filtered (cut-off frequency of 7 Hz) and interpolated. Kinematic parameters were automatically extracted by *ad hoc* algorithms developed in Matlab® ambient (Matlab 2015b, The MathWorks, Inc., Natick, Massachusetts, USA) and then checked by visual inspection. Steady state linear walking was described by spatio-temporal gait parameters: stride length, duration, and velocity (normalized to subject’s height), stance and double-support duration. Temporal parameters (i.e. stance and double-support) were time-normalized as a percentage of the stride duration. For each subject and condition, all variables have been averaged over the trials. With the same experimental setup, we also evaluated 11 healthy controls (9 males, 2 females; median age 60 years, range 50–66 years) matched for age and anthropometric measurements.

Differences in spatio-temporal gait parameters between controls and PD groups were analyzed with Steel-Dwass all pairs test. We used JMP software version 13 (SAS Institute Inc., Cary, North Carolina, USA).

### SPECT imaging

SPECT data acquisition and reconstruction has been previously described [42,43]. All patients performed a SPECT with FP-CIT to measure the dopamine reuptake transporter (DAT) density within three months before surgery (in the best medical and DBS treatment state). Scans were started 180 min after injection of 182.4±3.6 MBq of FP-CIT on a dual-headed integrated SPECT/CT system (Symbia T2; Siemens, Erlangen, Germany). SPECT data were spatially normalized onto a FP-CIT MNI-based template and volumes of interest (i.e. left and right caudate nucleus, left and right putamen, left and right striatum) as well as a reference region in the occipital cortex were defined using the volume of interest (VOI) of the automated anatomical labeling method [44]. The non-displaceable binding potential (BP_ND_) for caudate nucleus, putamen and whole striatum (for both hemispheres) was then assessed using average regional uptake values from VOI analysis and the occipital cortex as reference region [45]. Based on molecular imaging data, we identified the hemisphere with less (STN–) or more (STN+) dopaminergic innervation. We used the whole striatum, rather than its motor part (i.e. the putamen), as the boundaries between the putamen and the caudate nucleus are uncertain in SPECT images. Asymmetry indexes (AI) for striatal BP_ND_ were calculated using the formula:

$$\frac{\left(BP_{ND}striatum\;ipsilateral\;–\;BP_{ND}striatum\;contralateral\right)}{\left(BP_{ND}striatum\;ipsilateral\;+\;BP_{ND}striatum\;contralateral\right)}\;x\;200$$ (2)

Contralateral refers in this case to the striatum opposite to the clinically most affected side. For healthy subjects, we conventionally refer to the right side as ipsilateral [46,47]. Striatal DAT binding measurements of the patient group were compared with normal values of 15 healthy subjects (4 males, 11 females; median age 67 years, range 44–74). The Institutional Review Board of the Fondazione IRCCS Ca’ Granda Ospedale Maggiore Policlinico di Milano approved the investigation, which was conducted in accordance with the Declaration of Helsinki, and all patients gave written informed consent.

Differences in BP_ND_ of DAT between controls and PD groups were analyzed with the Steel-Dwass all pairs test. We used JMP software version 13 (SAS Institute Inc., Car, North Carolina, USA).

### LFPs recordings and synchronization

We recorded subthalamic LFPs with a single bipolar derivation for each STN, amplified by 2000 and sampled at 422 Hz. The recording contacts were selected accordingly to clinical effect of chronic stimulation: we selected a bipolar montage crossing the chronically active electrode [48].

A limitation of the Activa PC+S^®^ system is the recording of the ECG artefact by the ventral contacts [49]. We removed ECG waves in LFP recordings of three out of 14 STNs by detecting the signal peaks representing QRS complexes. Details about the ECG correction algorithms and relevant discussion can be found in [19].

Synchronicity across STN recordings and kinematic measures has been previously described [19]. In brief, synchronization was achieved by means of a transcutaneous electrical nerve stimulation (TENS) artefact that was introduced on-demand into the acquisition systems. The electrodes for the TENS were placed in the supraclavicular fossa, directly on the cable that connects the impulse generator with the electrodes, and over the burr-hole site. The same TENS artefact fed into the Activa PC+S^®^ and the SMART-DX400. We introduced two TENS artefacts: one at the beginning and one at the end of each recording session, thus accounting for the different nominal and real sampling frequencies of the acquisition systems. We detected the TENS artefact in all recordings and used the sharp drop-off as synchronization instant across modalities. After synchronization, the analyses were further performed with the dataset containing LFPs resampled at 400 Hz, excluding the time windows with the TENS artefacts.

### Power spectral analyses

**Fig 1:**
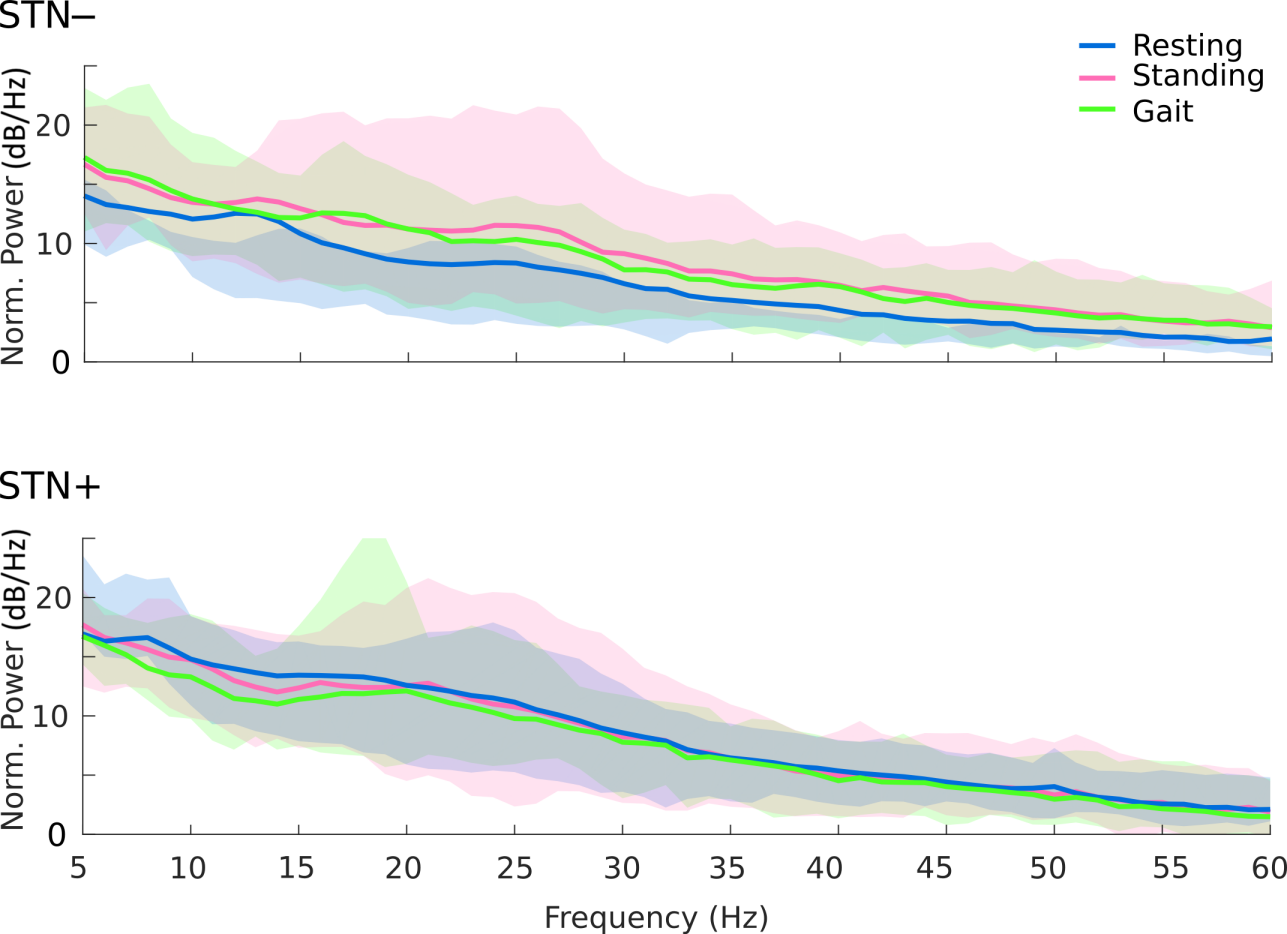
Spectral profiles (average) during resting, upright standing, and gait. Average spectral power of the STN local field potential during resting (blue line), standing (pink line) and gait (green line) for the two hemispheres, with less (–) and more (+) striatal dopamine innervation. Results are corrected for the nominal noise floor level of the device (150nV/rtHz, [32,33]), all values above 0dB/Hz are reliable. Shaded areas represent the confidence intervals (5–95%) of the group mean. The peak at 32 Hz is a known artefact of the Activa PC+S^®^ system tied to clock settings or due to a triggered check of the battery status.

We characterized the STN power modulation of resting, upright standing and walking by means of the multi tapers method (five tapers) [50]. Given the relative novelty of the Activa PC+S® system, we first defined the quality of the acquired signal with respect to the internal noise. Manufacturer datasheet as well as previous publications reported a nominal noise floor (NF) for the beta band of 150nV/rtHz [32,33]. We normalized the PSD dividing by the square of NF and transformed it into dB/Hz (10Log10(PSD/NF^2)). After this conversion, 0dB/Hz corresponds to the minimum threshold for reliability (Fig 1). We then estimated group level variance using the bootstrapping (20 repetitions; resampling with replacement) technique and estimated the confidence intervals between 5th and 95th percentiles of the bootstrap distributions.

We then analyzed the STN activity within the gait phases. We used the wavelet transform with 60 Morlet wavelets with 10 cycles/s (from 6 to 90 Hz) [51]. Frequencies below 6 Hz were discarded as the selected time resolution of 10 cycles/s exceeds the stride duration. This allowed exclusion of low-frequency biological artefacts. We calculated the event-related power relative changes (event related synchronization [ERS] and desynchronization [ERD]), normalizing the mean low-*β*, high-*β* and *γ* power by subtracting and dividing for the average of the entire recording (baseline), to reduce the effects of single-trial normalization:

$$\left(X-\bar{Baseline}\right)/\bar{Baseline}$$ (3)

Since stride length varied across subjects, we normalized the ERS and ERD of each stride to a reference stride duration by means of piece-wise polynomial time-warp algorithm, similarly to that proposed by Sadeghi and coll. [52]. Briefly, the algorithm performs a polynomial interpolation of the time axis against a reference period (the stride in our case). We used a third order polynomial interpolation with a 2 s time-window centered at each velocity peak (VP) of the swing foot. This time-window exceeded the duration of the stride and ensured that the border-effect did not influence the analysis window. We analyzed the STN activity (either STN–or STN+) contralateral to each foot VP (VP_contra_). A time-warp algorithm was applied for each single stride and stride phases (i.e. single- and double-support phases of the stance, acceleration and deceleration phases of the swing) defined according to the instants: heel contact, toe off and velocity peak of the swing foot.

### Network analyses

We investigated the inter-hemispheric connectivity of the two STNs by computing the spectral coherency (Coh), the phase locking value (PLV) [53] and the amplitude cross-correlation (CC). We selected this metric as they estimate the synchronized rhythmic excitability of neural ensembles, which is considered useful for the flow of information between nodes of a functional network as it might strengthen the information transmission [9,53,54]. The Coh represents one of the most widely accepted metric of functional connectivity [9]. The PLV and the CC disentangle the two main components of the Coh, respectively the phase and the amplitude correlation of the investigated signals [53,55].

We divided resting, standing and walking recordings in multiple epochs of 3 s and computed all the following interaction metrics in each epoch and averaged across epochs for each subject separately. For spectral coherence, we used multi-tapers spectral profiles computed over 13 frequency bins (2 up to 90 Hz) defined to minimize the overlaps and with constant relative bandwidth equal to 0.5.

Coherency is defined as:

$$Coh\left(X,Y\right)=\left|\frac{Sxy}{\sqrt{Sxx*Syy}}\right|$$ (4)

where *Sxx*, *Syy* represent the auto-spectrum of signal X and Y, respectively and *Sxy* represents the cross-spectrum. This measure (i.e. Coh) is dependent from both phase and amplitude, which were further separately measured. We band-pass filtered the (artefact free) LFPs with 13 finite impulse response (FIR) filters with central frequencies 2 up to 90 Hz and with constant relative bandwidth of 0.5. The band-pass filtered data were then Hilbert transformed to extract the analytic signal

$$\hat{X}\left(t\right)=H\left(X\left(t\right)\right).$$ (5)

The PLV is defined as:

$$PLV\left(X,Y\right)=\;\frac{1}{N}\left|\sum_{i}^{N}e^{j\Delta \varphi_{i}}\right|$$ (6)

where Δφ_i_ represents the phase difference between X and Y at the i-th time sample. The PLV describes the phase of the signals, being independent from their amplitude, and it helps understanding the Coh, especially when the signal-to-noise ratio of the recording is questioned [55].

We then computed the correlation of the signals amplitude with the CC analysis. To quantify CC between amplitudes

$$X_{a}=\left|\hat{X}\right|$$ (7)

of signal *X* and *Y* we used the Pearson correlation coefficient (*r*) defined as:

$$r\left(X,Y\right)=\;\frac{1}{N}\sum_{i}^{N}\frac{X_{a}-\mu_{X_{a}}}{s_{X_{a}}}\frac{Y_{a}-\mu_{Y_{a}}}{s_{Y_{a}}}$$ (8)

where *(μ)* represent the sample mean and *(s)* the sample standard deviation.

All three connectivity measures range from 0 to 1, meaning “no” or “complete” coherence, phase synchrony or CC, respectively. For these measures, we quantified surrogate noise levels by time-rotation of the original time series. Specifically, under the null-hypothesis of no correlation between signals *x* and *y*, we split-and-rotate the time course of *y* at random onsets and quantified all three connectivity metrics on the time-rotated *y* against the original signal of *x*. We repeated this procedure 100 times, assessing the distribution of the null-hypothesis. For each of the three metrics separately, we computed the *p-*value as the number of elements from the surrogate distribution that exceeded the observed measure. We considered only significant values (*p*<0.05) for further statistical analyses. As for power modulation analyses, we quantified the group level variance using similar bootstrapping approach. We compared all possible combinations (i.e. rest vs. walking, standing vs. walking, rest vs. standing) for all the computed indices by means of the Wilcoxon test matched pairs. Of relevant note, we were not able to compare inter-hemispheric connectivity by gait phases because of the overlay between the two hemibodies (i.e. the single-support phase of one leg is the swing phase of the other leg, and vice versa).

We instead investigated the correlation between inter-hemispheric connectivity and striatal dopaminergic denervation. To this end, we estimated the overall striatal dopaminergic loss, which was calculated as the mean of the dopaminergic loss of left and right striatum. However, this measure prevented the distinction between hemispheres (as for PSD), only illustrating the overall dopaminergic loss of each subject. To overcome this limitation, we also correlated the PLV with the AI (Eq 2) as a measure for the dopaminergic degeneration imbalance between the two hemispheres.

All methods and analyses were implemented in Matlab (Matlab 2013a, The MathWorks, Inc., Natick, Massachusetts, USA) using *ad hoc* algorithms, fieldtrip [56] and integrated in Brainstorm [57]. Due to strict privacy laws, the datasets and analyses of this study shall be made available only upon personal request.

## Results

### Clinical data

Before surgery (pre-DBS), the median UPDRS-III score in meds-off condition was 44 points (range: 26–55) and 11 points (range: 4–24) in meds-on condition. After surgery (post-DBS), the median UPDRS-III score in meds-off and stim-off condition was 42 points (range: 27–65), in stim-on (and meds-off) condition was 15 points (range: 9–31), in meds-on (and stim-off) was 12 points (range: 8–23), and in meds-on and stim-on condition was 10 points (range: 5–16). The median percentage of improvement (see formula in *Patients and surgery* section) due to dopaminergic medication was 76.79% (range: 42.50–92.73%) and the one related to STN-DBS was 66.56% (range: 52.50–83.64%). Complete demographic and clinical information of each subject are listed in Table 1.

**Table 1:** Clinical data.

Subject	Age (years)	Disease duration (years)	LEDD (mg)		UPDRS-III (score)					
			Pre*-*DBS	Post*-*DBS	Pre-DBS		Post-DBS			
					Meds-off	Meds-on	Meds-off stim-off	Meds-off stim-on	Meds-on stim-off	Meds-on stim-on
wue03	61	18	2725	600	40	9	45	17	23	14
wue09	55	19	1200	730	50	11	33	16	8	11
wue04	54	7	658	400	26	8	27	5	9	8
wue02	65	10	1100	800	40	23	39	19	17	16
wue10	56	10	1200	550	69	14	65	25	20	5
wue07	61	10	650	220	43	24	29	15	8	9
wue06	51	11	1133	180	46	11	48	12	11	6
wue11	53	11	1300	460	55	4	51	9	13	14

Patients were evaluated with the UPDRS-III within one month prior to the implants (pre-DBS) after overnight (>12 h) suspension of all dopaminergic drugs (meds-off) and upon receiving 1 to 1.5-times (range 200–300 mg) the levodopa-equivalent of the morning dose (meds-on). After surgery (post-DBS), patients were also assessed with the UPDRS-III in four conditions: (i) stimulation off for at least two hours (stim-off); (ii) bilateral STN stimulation (stim-on); (iii) meds-on (as pre-DBS); (iv) meds-on and stim-on. Wue11 is the only female patient. DBS, deep brain stimulation; LEDD, levodopa equivalent daily dose; STN, subthalamic nucleus; UPDRS-III, Unified Parkinson Disease Rating.

### Biomechanical data

In agreement with previous studies, subjects with PD showed shortened stride length and lower stride velocities (both average and maximum velocity) with respect to matched healthy subjects [41,58]. Of note, the duration of the stance and double-support phase (as percentage of the stride) was within the normal range, thus suggesting that despite the disease-related biomechanical alterations, the patients preserved a normal timing of the gait cycle (i.e. no shuffling gait or festination) (Table 2).

**Table 2:** Anthropometric and kinematic data.

Subject and group	wue03	wue09	wue04	wue02	wue10	wue07	wue06	wue11	PD	HC
Body height (cm)	180.55	181.92	170.99	176.16	187.43	175.45	167.17	166.72	175.80±7.32	174.24±6.47
Inter-ASIS distance (mm)	284.63	287.64	236.48	329.34	273.9	238.92	252.84	260.74	270.56±30.54	290.07±34.74
Foot length (mm)	266.1	255.05	260.94	272.13	268.41	240.92	238.53	250.82	256.61±12.52	254.11±15.22
Limb length (mm)	935.36	936.8	892.24	854.05	938.24	915.47	875.85	863.11	901.39±34.65	900.43±29.62
Weight (Kg)	93.95	100.58	71.91	107.49	98.35	77.49	67.52	101.5	89.85±15.23	76.54±10.74
BMI (Kg/m^2^)	28.82	30.39	24.6	34.64	28	25.17	24.16	36.52	29.04±4.61	25.22±3.58
Stride duration (s)	1.18±0.05	1.08±0.03	1.23±0.03	1.18±0.02	1.16±0.04	1.09±0.04	1.1±0.04	1.18±0.04	1.15±0.05	1.13±0.09
Stance duration (%stride)	62.38±1.95	64.11±1.65	63.93±1.16	65.11±1.61	63.66±1.57	59.31±2.07	60.52±1.37	65.55±1.57	63.07±2.19	62.31±1.62
Double-support duration (%stride)	24.11±1.97	28.24±2.59	27.97±1.69	30.55±1.75	27.07±2.22	18.22±3.41	21.22±1.94	31.03±2.48	26.05±4.52	24.58±3.32
Stride length (%BH)	52.23 ±0.04	62.03±0.06	61.99±0.03	72.38±0.04	66.12±0.07	53.32±0.06	64.56±0.09	54.58±0.05	60.90±7.04*	72.00±6.41*
Stride average velocity (%BH/s)	44.26±0.05	52.57±0.05	50.40±0.04	65.80±0.06	60.66±0.05	49.37±0.07	55.65±0.07	46.26±0.05	53.12±7.28*	64.17±9.37*
Stride max velocity (%BH/s)	147.59±0.25	165.61±0.7	163.17±0.13	195.01±0.15	181.25±0.19	166.56±0.20	187.8±0.20	151.75±0.13	169.84±16.83*	199.63±21.44*

Stance and double-support duration are expressed as the percentage of the duration of the stride (i.e. the interval between two subsequent heel strikes of the same foot). The stride length and the stride average velocity were calculated as a percentage of the body height (BH) of each subject. Data are shown as mean ± standard deviation.

* *p*<0.05

Steel-Dwass all pairs. ASIS = anterior-superior iliac spines; BMI = body mass index; HC = healthy control group; PD = Parkinson’s disease patient group.

### SPECT data

As expected, all PD patients showed significantly reduced non-displaceable binding potential (BP_ND_) of DAT in the caudate nucleus and the putamen. The striatal dopaminergic loss exceeded 50% bilaterally in six out of eight subjects (Table 3). The clinically most affected side (higher UPDRS-III scores) always corresponded to the striatum with less nigro-striatal dopaminergic innervation. The most dopamine-depleted hemisphere was the right one in three subjects.

**Table 3:** Non-displaceable binding potentials of dopamine reuptake transporters.

Subject and group	Caudate nucleus left	Putamen left	Striatum left	Caudate nucleus right	Putamen right	Striatum right	Asymmetry index	STN–
wue03	0.49	0.31	0.40	0.31	0.28	0.30	28.57	R
wue09	0.72	0.48	0.61	0.62	0.37	0.49	21.82	R
wue04	1.15	0.70	0.93	0.58	0.44	0.50	60.14	R
wue02	0.88	0.57	0.72	1.43	0.91	1.17	47.62	L
wue10	0.97	0.50	0.75	1.20	0.74	0.96	24.56	L
wue07	0.92	0.64	0.76	1.22	0.79	1.00	27.27	L
wue06	1.31	0.59	0.95	1.56	0.91	1.20	23.26	L
wue11	1.15	0.79	0.96	1.41	0.74	1.05	8.96	L
PD	0.95±0.26*	0.57±0.15*	0.76±0.19*	1.04±0.47*	0.65±0.25*	0.83±0.35*	30.27±16.09*	
HC	2.57±0.57	2.30±0.42	2.33±0.48	2.62±0.52	2.23±0.48	2.30±0.48	2.57±2.14	

The BP_ND_ of DAT in the striatum was used to identify the hemisphere with less (i.e. STN–, bold) or more (i.e. STN+) dopaminergic innervation. For healthy subjects, we considered the average striatal binding of left and right, which did not significantly differ. Data are shown as mean ± standard deviation.

* *p*<0.05

Steel-Dwass all pairs. BP_ND_, non-displaceable binding potential; DAT, dopamine reuptake transporter; HC = healthy control group; PD = Parkinson’s disease patient group; STN = subthalamic nucleus.

### Local subthalamic oscillations

**Fig 2:**
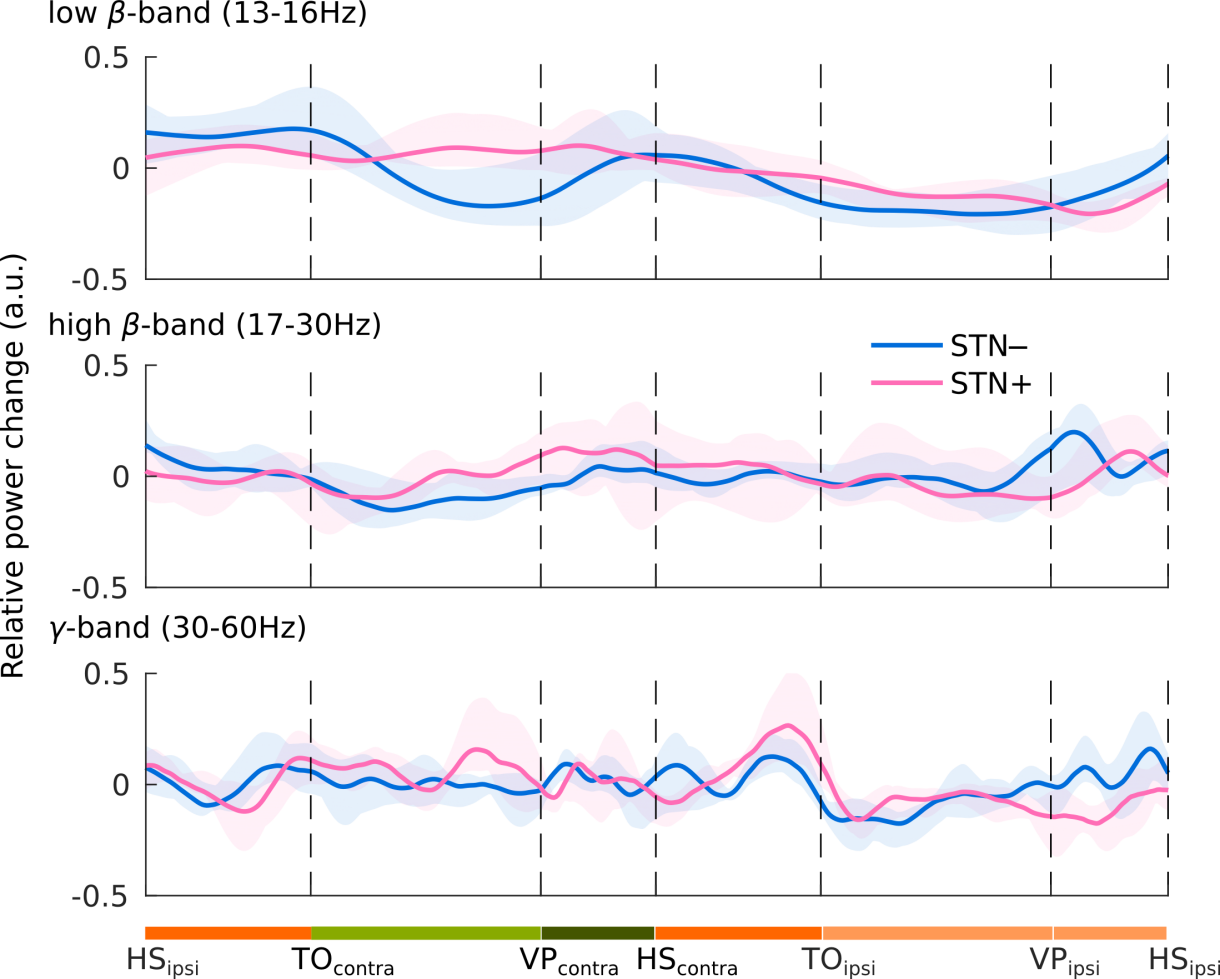
Modulation of the spectral power during the gait cycle. Event related synchronization (ERS) and desynchronization (ERD) in low *β-* (top) high *β-* (middle) and *γ*-frequency band (bottom). Subthalamic power changes of the phases of gait are shown as the average relative change of the whole stride of all subjects. Shaded areas represent the confidence intervals (5–95%) of the group mean. We analyzed the power changes of STN–and STN+ during the gait cycle of the contralateral foot (but they could be also referred to the matched gait phases of the ipsilateral one). Stance is the period during which the foot is on the ground (dark and light orange bars). The stance phase includes a period of bilateral foot contact with the floor (double-support phases [dark orange bars]), and a period of unilateral foot contact (single-support phase [light orange bar]). The swing phase (light green and dark green bars) is the interval in which the foot is lifted from the floor. Thanks to the velocity peak (VP) of the marker placed on the lateral malleolus, we identified an acceleration (light green) a deceleration (dark green) sub-phase of the swing phase. HS = heel strike; TO = toe off; VP = velocity peak; lower case subscript indicates the foot contralateral _(contra)_ or ipsilateral _(ipsi)_ to STN–or STN+.

**Fig 3:**
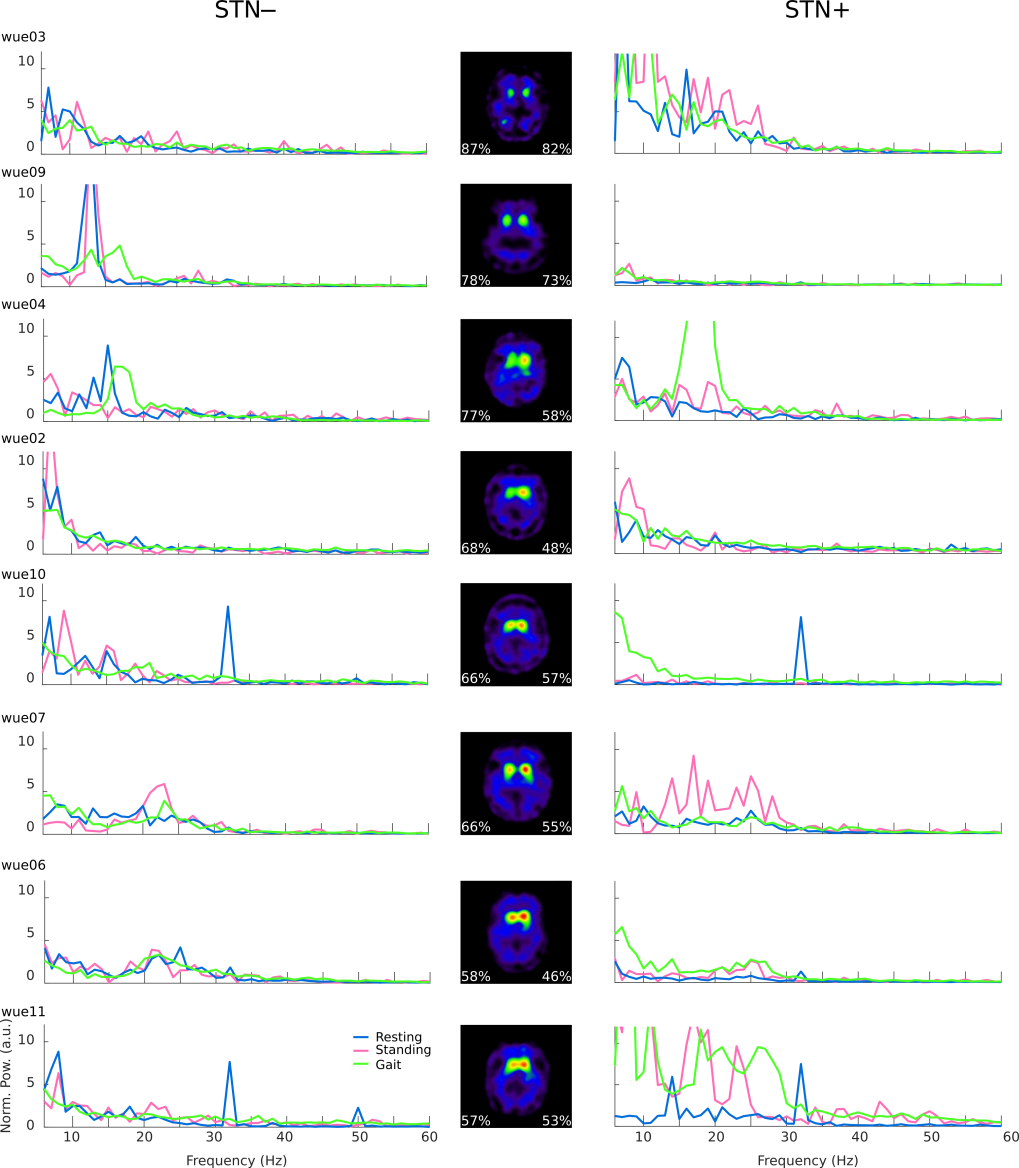
Spectral profiles (single subject) during resting, upright standing and gait. Single subject spectral power of the STN local field potential during resting (blue line), standing (pink line) and gait for the two hemispheres, with less (–) and more (+) striatal dopamine innervation. Axial slices are left-right flipped to match the corresponding STN. The peak at 32 Hz is a known artefact of the Activa PC+S^®^ system tied to clock settings or due to a triggered check of the battery status. SPECT scans (central column) show striatal dopaminergic loss as percentage decline with respect to healthy subjects (calculated from BP_ND_ of DAT, Table 3).

We did not find any clear modulation of local STN activity when comparing the power profiles of walking and standing with resting state (Fig 1). The STN activity was also not modulated during specific gait phases (Fig 2). The STN neuronal oscillations were not related to the striatal dopamine content, despite the wide range of DAT binding loss (46–86%) in our patient collective (Fig 3). Still, the STN+ showed an increase of the average *β*-band power during walking, which did not reach a statistical significance (Fig 1). The lack of a clear pattern was particularly evident when looking at each STN independently. We showed a bilateral reduction of the *β*-activity during walking in five STNs (of four subjects: wue09 STN–, wue10 STN–, wue07 STN–and STN+, wue03 STN+), whereas five STNs (of four subjects: wue04 STN–and STN+, wue10 STN+, wue6 STN+ and wue11 STN+) showed an increase of the *β*-power (Fig 3).

### Inter-hemispheric subthalamic coupling

**Fig 4:**
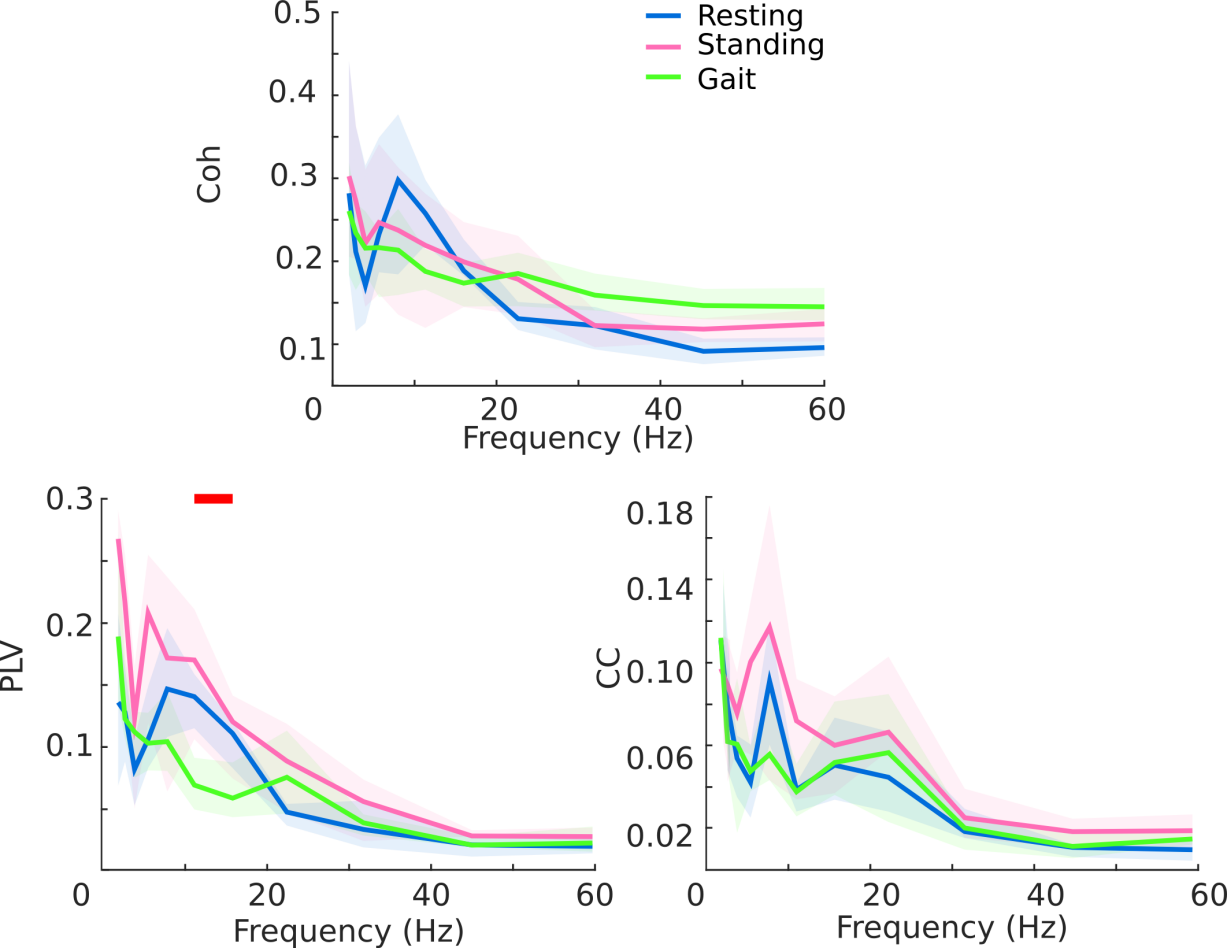
Inter-hemispheric coherence. Inter-hemispheric coherence (Coh, top panel), phase locking value (PLV, bottom left plot) and amplitude cross-correlation (CC, bottom right plot) during resting state (blue line), upright standing (pink line) and walking (green line). Statistical significance (red bar, paired Wilcoxon test, *p*<0.05 uncorrected) was reached for the PLV selectively in the *β*-frequency band between resting state and walking. Shaded areas represent the confidence intervals (5–95%) of the group mean.

We showed a reduction in the inter-hemispheric subthalamic coupling during walking with respect to the upright standing and resting state condition. In particular, the phase synchronization (PLV) of the two STNs diminished significantly in the *β*-frequency band (Fig 4). We did not find any correlation between the PLV values and the overall striatal dopaminergic loss (resting: ρ: 0.47, p = 0.24; standing: ρ: 0.19, p = 0.65; walking: ρ: 0.05, p = 0.90) or the AI (resting: ρ: -0.61, p = 0.1; standing: ρ: 0.32, p = 0.44; walking: ρ: 0.35, p = 0.40).

## Discussion

In this study, we showed in PD patients an inter-hemispheric subthalamic coupling in the *β-*frequency band during resting and standing, and a desynchronization during gait (Fig 4).

The framework of human locomotion control starts in the SMA, reaches the basal ganglia for refinement and then the MLR, where the cerebellar inputs converge, to descend to the medullary and pontine reticular formations and the spinal cord [59]. The STN is a cornerstone of this network, receiving direct afferences from the SMA and projecting to both the MLR and the basal ganglia output nuclei (i.e. the GPi and the SNr), which also project to the MLR [15]. The STN modulates the integration of cortical and cerebellar information by activating or inhibiting the MLR via direct glutamatergic projection or basal ganglia GABAergic output [59]. These *feed-forward* pathways can support the information processing in the MLR and the medial reticular formation (i.e. rhythm generating system)[59], which projects to the CPGs in the spinal cord.

We suggest that subthalamic inter-hemispheric desynchronization may reflect the downstream conveying of locomotor information for each body side separately, which may facilitate the spontaneous alternating activity of the spinal CPGs for steady linear walking. Of relevance, the movement-related modulation of the subthalamic coupling was narrowed to the *β-*frequency band (Fig 4). This result further supports the relevance of *β* oscillations in motor control. The functional circuits of the brain are multiple and segregated by frequency, so that the precise tuning to distinct frequencies could provide a way of marking and segregating different motor networks, over and above any anatomical segregation of processing streams [60].

Besides this physiological interpretation, we cannot exclude that the changes in subthalamic dynamics during gait in PD may be compensatory or maladaptive. The lack of correlation between the PLV and the overall striatal dopaminergic loss and the AI argues against this interpretation. However, it is well known that in the untreated state, PD is dominated by pathologically exaggerated synchronization and coherence in the basal ganglia-cortical circuit, which implies a shift from a dynamic system to stability [17,38,61–67]. Therefore, the subthalamic inter-hemispheric desynchronization during gait, with respect to resting and standing, might also reflect a need to compensate for an excessive static connectivity (persistent *β-*coupling) of the motor network.

Overall, these findings are of particular interest considering the evolution of stimulation therapy towards (adaptive) closed-loop systems directly triggered by biological signals [29]. By timing the stimulation bursts to the phase of tremor oscillations, Cagnan and coll. showed that phase-specific stimulation could control tremor severity in subjects with essential tremor and dystonia [68]. In this study, the stimulation was triggered by kinematic measurements of tremor, but the *trigger* could be easily extended to recordings of the LFP, using the strength of different oscillators to determine the stimulation phase in real-time [68]. During the development of such an applicable closed-loop stimulation, besides identifying reliable markers of a pathological brain activity it would be crucial to characterize the activity patterns of common and frequent motor behaviors (e.g. gait) [29]. Our study represents the first step in this direction.

Much of the interpretation of our findings presently remains speculative, given also the absolute novelty of our recordings. A subthalamic inter-hemispheric network has been consistently described and investigated in subjects with PD [19,22–25], but only during resting state or simple unilateral movements. In particular, voluntary movements modulate the subthalamic coupling in the *θ-* and *α-*frequency bands, but not in the *β-*frequency band [19,30]. This led to the hypothesis that the subthalamic coherence in the *β-*frequency band in PD could directly reflect a striatal dopamine loss [19], but its inconsistent suppression by levodopa challenges this idea [23]. Alternatively, inter-hemispheric synchronization and desynchronization in the *β-*frequency band may serve selectively complex and bilateral motor control processing [60], such as locomotion.

A secondary finding of our study is the inter-hemispheric subthalamic coupling during resting, which likely reflect polysynaptic inter-hemispheric neural interactions [22]. This result is in line with previous studies in PD [19,22–25] and complements the inter-hemispheric cortical coupling [64,69] and cortico-subthalamic coupling [64,70] in defying a long-distance synchronization network (*β-*network) in PD [22]. A number of anatomical connections may contribute to such a network, including (i) bilateral cortical inputs and synchronization via the corpus callosum [64,69–71] of the *hyperdirect* [72] or the *direct* pathways [15], (ii) bilateral projections of the striatum to both STNs [73], and (iii) subthalamic connections through the GPi/SNr and the MLR [23].

In contrast to network activity, we did not find a clear modulation of local subthalamic activity (i.e. PSD) during walking or standing with respect to resting state (Fig 1). This (unexpected) result is, however, in line with two previous studies that also failed to show a clear suppression of the *β-*band power in the STN of 15 PD patients during walking [49,74]. The lack of *β-*suppression in the STN during walking may be due to the small *β-*activity recorded in the recruited patients (Fig 3) and to the technical limitations of the recording device, which showed a poor signal-to-noise ratio. However, we have corrected our analysis for the nominal noise level of the device by normalizing each PSD to 150 muV/Hz [32,33] and we were able to capture with the same device a clear subthalamic *β-*suppression during upper-arm movements in three patients of the current sample (i.e. wue02, wue09, and wue11) [19]. It seems therefore unlikely that the lack of consistent *β-*suppression relies on device limitations. Instead, it should be considered that in this study we did not assess the subthalamic *β*-activity during transition phases (i.e. when changing an *ongoing* motor action, such as at gait initiation or termination), and the subthalamic *β*-activity signaled the likelihood of a forthcoming motor or cognitive action [75–77]. Therefore, the sensorimotor systems in PD could maintain a *status quo* (lack of *β-*suppression) during standing and unperturbed walking as steady state motor actions.

The mismatch between local and network activity, and the lack of a correlation with striatal dopamine loss (Fig 3), might explain the difficulties in ameliorating gait disturbances with STN-DBS as well as the poor levodopa responsiveness of some of these symptoms [78].

Despite these negative findings, we would like to report an anecdotal subthalamic *β-*suppression during the swing phase of the gait cycle (Fig 2, epochs between TO and HS [ipsi- and contralateral]). This *β-*suppression was limited to the STN, thus possibly reflecting the striatal dopamine influence of movement control [19].

This study has several limitations. Firstly, without histological verification the placement of the electrodes in STN remains presumptive. Secondly, although our analytical techniques are biased against the detection of stochastic, non-oscillatory activity, we cannot exclude having captured such oscillations (that characterize the ventro-medial area of the STN) in our analysis. Still, we verified the electrode location by fusing the pre- and post-operative imaging of each subject and we recorded from the chronically-active electrodes, which were effective in improving motor symptoms (Table 1). Furthermore, none of the patients reported stimulation-induced behavioral or mood changes related to ventral STN stimulation, thus indirectly confirming that we recorded the activity of the dorsolateral part of the STN, the area with predominant oscillatory activity [79–82]. Thirdly, the subthalamic coherence can be due to the volume conduction of a synchronous activity from a third source (e.g. the cerebral cortex). Measurements of coherence are indeed susceptible to zero-phase volume conduction effects; however, this should not be the case for inter-hemispheric interactions. Previous studies demonstrated that the extent of the volume conduction effect depends on several issues such as the source density, the orientation, and the conducting media [83]. This led to a wide range of possible distances (from 0.6 to 5 mm) at which volume conduction may occur [83], but all below the distance between the two STNs. The distance across STNs saved our recordings from the common input of the background magnetic field (i.e. volume conduction), but did not prevent the possible interference from a third (unknown) source of oscillations, which might influence the synchronization analysis. Although the STN afferents are mainly ipsilateral, studies in rats have identified a few thalamic and peduncolopontine axons that also projected to the contralateral STN [84,85].

Despite these limitations, it is worth mentioning that we recorded the LFPs months after surgery and with a fully implanted device, thus limiting the influence of the high impedance variability and the microlesioning effect that bias the immediate post-operative recordings [70].

In summary, this study highlights the inter-hemispheric subthalamic desynchronization in the *β-*frequency band during walking in subjects with PD. This result has a twofold bearing. First, it expands our understanding of locomotor control in subjects with PD. This topic is of particular relevance given the high incidence of gait disturbances in these patients and the related consequences (i.e. falls, fractures, institutionalization, loss of independence, and increased mortality) [86]. Second, this study suggests that in some cases (i.e. gait), the brain signals, which could allow feedback-controlled stimulation techniques, might derive from an inter-hemispheric network activity rather than local oscillations (e.g. single STN beta-band activity) [74].
